# P088: Monitoring multiresistant bacteria (MRB) to Principal Hospital Dakar: assessment of 1 year

**DOI:** 10.1186/2047-2994-2-S1-P88

**Published:** 2013-06-20

**Authors:** B Fall, B Wade, B Niang, MN Seye, E Dieme, KB Fall, KS Ndiaye, S Diawara, SB Gning, Y Dieme

**Affiliations:** 1 Laboratories Federation, Dakar, Senegal; 2 Directorate, HPD, Dakar, Senegal; 3 Reanimation Department, HPD, Dakar, Senegal; 4 Pediatrics, HPD, Dakar, Senegal; 5 Surgical Services, HPD, Dakar, Senegal; 6 Medical Services, HPD, Dakar, Senegal

## Introduction

Bacterial resistance to antibiotics is a public health problem. Mastering their distribution is thus a priority. Thus, at the Principal Hospital of Dakar (HPD), a system for collecting and analyzing data of resistance has been established within the Committee against nosocomial infections.

## Objectives

We present here the results compiled over a year to help guide prevention activities.

## Methods

Prospective study from January 1 to December 31, 2012 at the HPD. Every day, multiresistant bacteria isolated in the laboratory are subject to a collection of clinical and biological data using a questionnaire. Enterobacteriaceae producing extended-spectrum beta-lactamase (ESBL) and derepressed cephalosporinases of Pseudomonas aeruginosa, multiresistant Acinetobacter and Methicillin Resistant Staphylococcus aureus isolates were analyzed. The data are then analyzed by Epi info.

## Results

323 BMR were collected during the study period. The average age was 32 years [4 days, 95 years] and the sex ratio was 1.70. ESBL-producing Enterobacteriaceae (80%) followed by Acinetobacter multiresistant respectively (11%), ticarcillin-resistant Pseudomonas aeruginosa (4%) and methicillin-resistant Staphylococcus aureus (4%) were the most common isolates. ESBLs were as follows: 55% Klebsiella, E. coli 32% 11% Enterobacter, and others 2%. Blood cultures were the most common samples (40%), followed respectively by urinary tract infections (37%) and abscesses (16%). The pediatrics department was most affected (45%), followed respectively by the Internal Medicine and Resuscitation (each 23%) and Surgery (9%). A catheter was present in 91% of patients with sepsis and 66% of ESBL infections ESBL-producing Enterobacteriaceae were considered nosocomial.

## Conclusion

This study shows the important place occupied by multi-resistant bacteria Principal Hospital. ESBL-producing Enterobacteriaceae represent the most common resistant organisms, mainly in the form of nosocomial infections.

## Disclosure of interest

None declared

